# The International Hahnemannian Association

**Published:** 1896-07

**Authors:** 


### THE INTERNATIONAL HAHNEMANNIAN ASSOCIATION.

The seventeenth annual meeting of this Association was held at Glen Summit, Pa., on the 24th and 25th days of June. The meeting was the smallest in the history of the Association. This should not be. The Association should not be allowed to decline in interest and importance by reason of small attendance and scanty contributions of papers. There was a well-founded belief that new causes of dissension were to be brought before the Association, and this had the effect of keeping many members away. Notwithstanding the small attendance, the papers actually submitted and the discussions upon them were of the highest interest.

The address of the President, Dr. B. Fincke, was unanimously spoken of as a most scholarly and instructive paper. We hope to be able to reproduce it in full in the pages of The Homœopathic Physician.

After the usual reports of officers the regular business of the meeting was opened by the reports of the chairmen of the various bureaus. The following are some of the papers, which were interesting:

In the Bureau of Homœopathic Philosophy, of which Dr. George H. Clark was chairman, several papers of value were noticeable. "Harmonics of Homœopathics" was the title of one by Dr. J. H. Allen, of Logansport, Ind.

Dr. Fincke contributed a translation of a paper entitled, "On the Appreciation of High Potencies," by Dr. C. Von Bœnninghausen, in Munster. This paper was highly interesting. Dr. A. McNeil, of San Francisco, contributed a paper on the "Genius Epidemicus." Dr. C. H. Oakes contributed an intensely sarcastic and very witty paper entitled, "Is Consumption Contagious?"

This paper heaped ridicule upon the dominant school of medicine and was very entertaining.

